# Pterostilbene, a natural phenolic compound, synergizes the antineoplastic effects of megestrol acetate in endometrial cancer

**DOI:** 10.1038/s41598-017-12922-2

**Published:** 2017-10-06

**Authors:** Wei Wen, Gina Lowe, Cai M. Roberts, James Finlay, Ernest S. Han, Carlotta A. Glackin, Thanh H. Dellinger

**Affiliations:** 1 Department of Surgery, Division of Gynecologic Oncology, City of Hope Comprehensive Cancer Center, Duarte, CA 91010 USA; 2 Department of Developmental and Stem Cell Biology, Beckman Research Institute, Duarte, CA91010 USA; 3 Department of Obstetrics and Gynecology, Yale University, New Haven, CT06510 USA; 4 Department of Animal Resources, University of Southern California, Los Angeles, CA 90033 USA

## Abstract

Endometrial cancer is the most common gynecologic cancer in the United States and its incidence and mortality has been rising over the past decade. Few treatment options are available for patients with advanced and recurring endometrial cancers. Novel therapies, which are frequently toxic, are difficult to establish in this patient population which tends to be older and plagued by comorbidities such as diabetes mellitus and hypertension. Therefore, novel, non-toxic therapies are urgently needed. Megestrol acetate is a frequently used drug in endometrial cancer patients. However, its response rate is only 20–30%. To enhance the activity of megestrol acetate in endometrial cancer patients, we explored the potential of combining natural supplements with megestrol acetate and found that the addition of the natural phenolic compound, pterostilbene, to megestrol acetate resulted in a synergistic inhibition of cancer cell growth *in vitro* and an enhanced reduction of tumor growth in a xenograft mouse model. In addition, dual treatment led to attenuation of signaling pathways, as well as cell cycle and survival pathways. Our results demonstrated for the first time that the anti-tumor activity of megestrol acetate can be enhanced by combining with pterostilbene, providing an insight into the potential application of pterostilbene and megestrol acetate combination for the treatment of endometrial cancer.

## Introduction

Endometrial cancer is the most common gynecologic cancer in the United States, and unlike most other cancers, its incidence and mortality has been rising over the past decade^1–3^. While frequently curable in the early stage of this disease, a substantial portion of patients are diagnosed with incurable, advanced stage and recurrent disease. Additionally, endometrial cancer patients are often plagued by comorbidities such as obesity, diabetes mellitus, and hypertension, making novel therapies, which are frequently toxic, challenging to study. Only few treatment options are available for patients with advanced and recurrent endometrial cancer, and few novel drugs have been recently tested in clinical trial, with modest response rates^4–12^. One frequently used drug in endometrial cancer patients, especially those with metastatic lung lesions or who are deemed medically unfit for surgical management, is the progestin, megestrol acetate, which is associated with a 20-30% response rate in patients with advanced and recurring endometrial cancer^13–16^. To potentially enhance the activity of megestrol acetate in endometrial cancer patients, we have explored non-toxic natural supplements. Recently, the resveratrol analog, pterostilbene, a naturally occurring phenolic compound primarily found in blueberries, has been shown to possess antitumor activity^17–30^. Pterostilbene (PTE) has superior bioavailability as compared to resveratrol, with a favorable safety profile, and appears to act via apoptotic and anti-proliferative mechanisms in multiple solid cancer cells^17,18,31^. Specifically, its effects on cell death and cell cycle alterations have been documented in bladder, lung, and gastric cancer^32–34^. Recent reports suggest that its antioxidant and anticancer effects are mediated by estrogen receptors, as reported in breast cancer and colon cancer^32,35^. To date, the antitumor effects of pterostilbene have not been studied in endometrial cancer, a common estrogen-responsive cancer. We therefore hypothesized that pterostilbene would effectively reduce endometrial cancer growth both *in vitro* and *in vivo*, and enhance the antitumor activity of megestrol acetate in endometrial cancer. We tested the antiproliferative effect of pterostilbene with and without megestrol acetate in multiple endometrial cancer cells, and their anti-tumor effect in an endometrial cancer xenograft mouse model, while elucidating their effect on multiple growth and survival pathways, including JAK/STAT3, MAPK/ERK and PI3K/AKT pathways. Our results introduce pterostilbene as a potential therapeutic adjunct which effectively synergizes the antineoplastic effects of megestrol acetate in endometrial cancer, likely by reducing estrogen receptor expression, inhibiting STAT3 and MAPK/ERK signaling and subsequently suppressing cancer cell growth and survival.

## Results

### Pterostilbene inhibits endometrial cancer cell growth

**Figure 1:**
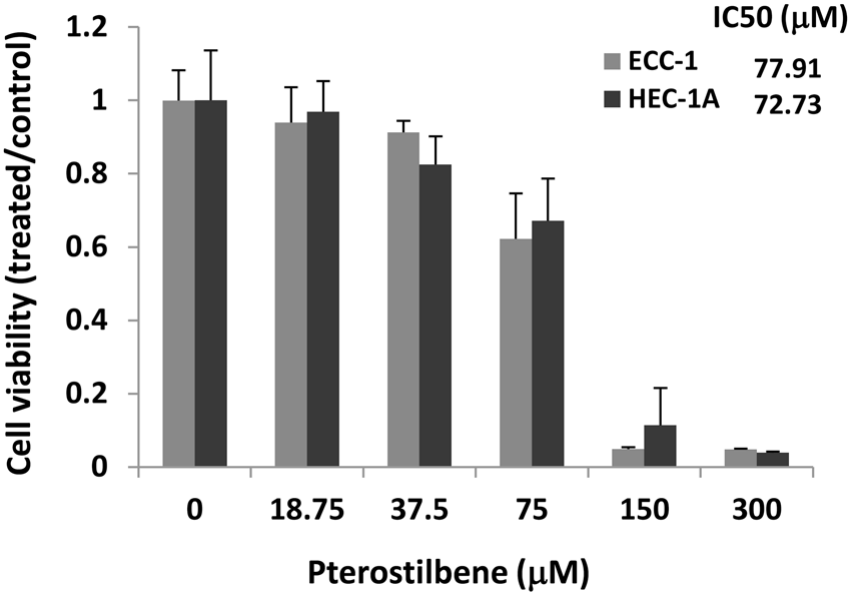
Pterostilbene (PTE) inhibits endometrial cancer cell viability. Cells were treated with vehicle (DMSO), pterostilbene (37.5–300 μM) for 48 hrs. Cell viability was determined using MTS assay. The IC_50_ was determined by the Chou-Talalay method. Data are expressed as the ratio to control treated with vehicle (DMSO).

To study the anti-tumor activity of pterostilbene in endometrial cancer, we tested its effect on cell growth in two endometrial cancer cell lines, ECC-1, an ER/PR responsive cell line derived from a patient with a well-differentiated endometrial cancer, and HEC-1A, a cell line derived from an endometrial cancer patient with adenosquamous histology with moderate ER-α expression^36–38^. Exponentially growing cells were treated with increasing concentrations of pterostilbene (37.5–300 μm) for 48 h. As shown in Fig. 1, pterostilbene significantly reduced cell viability in a dose-dependent manner, with IC_50_ (concentration for 50% growth inhibition) between 72 and 78 μM. These results indicate that pterostilbene can potently inhibit endometrial cancer cell growth.

### Synergistic effects of pterostilbene in combination with megestrol acetate

**Figure 2:**
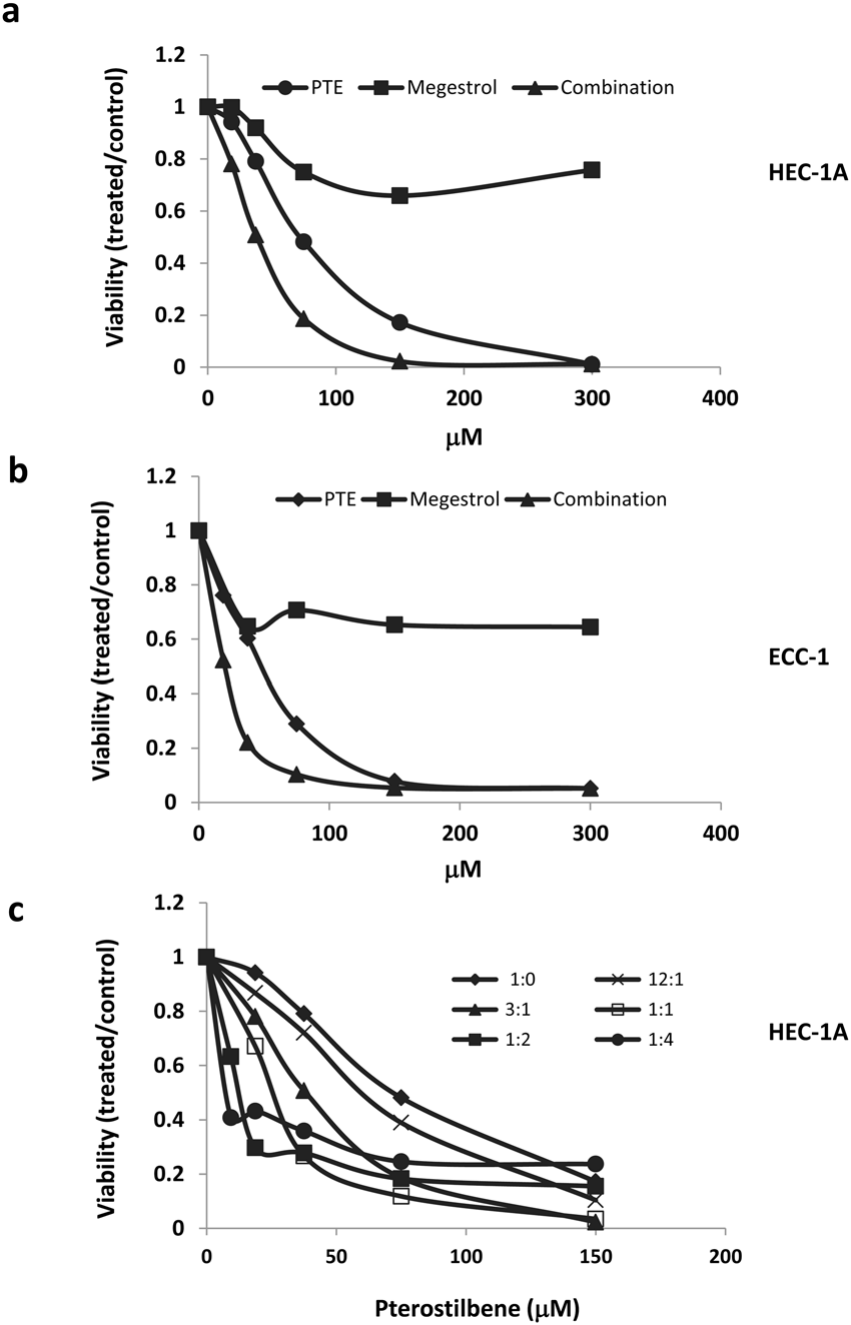
Synergistic effects of pterostilbene (PTE) in combination with megestrol acetate in human endometrial cancer cells. HEC-1A (**a**) and ECC-1 (**b**) cells were treated with pterostilbene or megestrol acetate either alone or in combination at various concentrations in a fixed molar ratio 1:1. Cell viability was determined 72 h later. (**c**) HEC-1A cells were treated with pterostilbene or megestrol acetate alone or in combination at various molar ratios. Cell viability was determined 72 h later. Results are representative of 3 or more preparations.

To study the effect of adding pterostilbene to megestrol acetate (Megace), we tested its effect on cell growth in the two endometrial cancer cell lines, ECC-1 and HEC-1A, either alone or in combination at various concentrations in a fixed molar ratio 1:1. Cell viability was determined 72 hours later (Fig. 2a,b). The combination index (CI) was determined using the Chou-Talalay method (CI = 1, additive effect; CI < 1, synergism; CI > 1, antagonism)^39^. Evaluation of the synergistic interaction revealed a positive synergistic effect for the combination of pterostilbene and megestrol acetate in both ECC-1 and HEC-1A cells, as shown in Table 1. The synergistic interaction between pterostilbene and megestrol acetate in HEC-1A is additionally depicted in a variety of molar ratios in Fig. 2c and Table 2. The combination treatment produced a strong synergism at each molar ratio. But it appears the combination at 1:1 molar ratio produced stronger synergy and a lower IC_50_ for both agents in the HEC-1A cells.

**Table 1:** Evaluation of synergistic interaction between pterotilbene (PTE) and megestrol acetate (Megestrol) in HEC-1A and ECC-1 cells.

Cells	PTE: Megestrol	Combination index (CI)			Fold reduction (IC_50_)	
		ED50	ED75	ED90	PTE	Megestrol
HEC-1A	1:1	0.36	0.47	0.61	2.75	>1000
ECC-1	1:1	0.34	0.47	0.64	2.91	>1000

**Table 2:** Evaluation of synergistic interaction between pterotilbene (PTE) and megestrol acetate (Megestrol) in variety of molar ratios on the viability of HEC-1A cells.

PTE: Megestrol	Combination Index (CI)			IC_50_ (μM)	
	ED50	ED75	ED90	PTE	Megestrol
12:1	0.86	0.89	0.93	51.66	4.30
3:1	0.71	0.72	0.74	36.57	12.19
1:1	0.36	0.47	0.61	23.30	23.30
1:2	0.17	0.50	1.43	11.17	22.34
1:4	0.08	1.16	16.8	5.10	20.40

### Combination treatment of pterostilbene and megestrol acetate suppresses cell survival and cell cycle pathways in endometrial cancer cells

**Figure 3:**
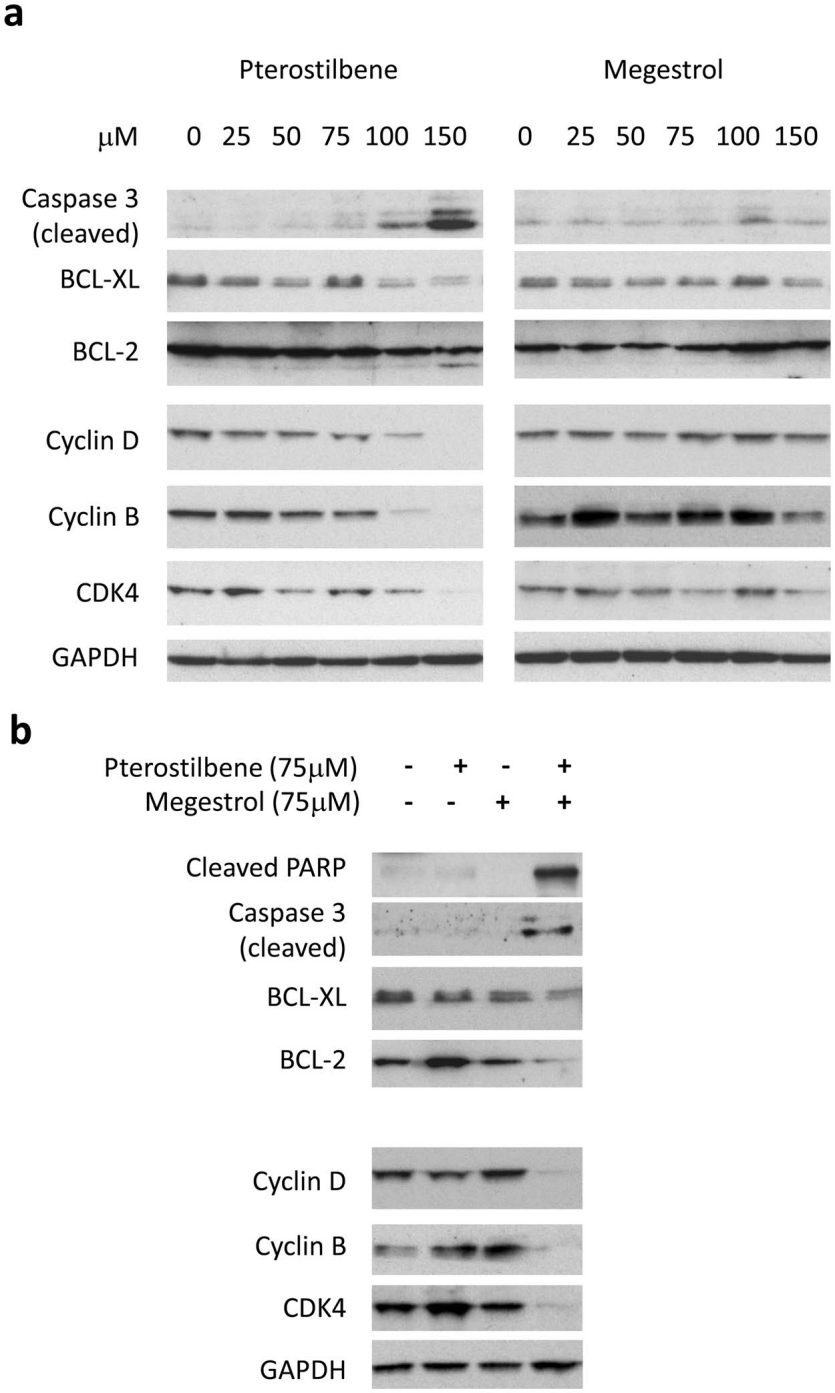
Effect of pterostilbene (PTE) and megestrol acetate (megestrol) on the expression of cell cycle molecules and cell survival molecules. (**a**) HEC-1A cells were treated with pterostilbene or megestrol acetate at various concentrations for 24 h. Whole cells were collected and determined for the expression of cell survival molecules and cell cycle molecules by Western blot. (**b**) HEC-1A cells were treated with pterostilbene (75 μM), megestrol acetate (75 μM) or the combination for 24 h. Whole cell lysates were collected and measured for the change of cell cycle and apoptosis pathways by Western blot. Results are representative of 3 or more preparations.

We next investigated the effect of combined treatment on the expression of proteins involved in cell survival. The administration of pterostilbene by itself caused increased cleavage of caspase 3, a molecular marker for apoptosis, and a decrease in BCL-2 and BCL-xl, two proteins for cell survival, in a dose dependent manner (Fig. 3a). megestrol acetate alone had little effect on the expression of these proteins (Fig. 3a). Combination of pterostilbene and megestrol acetate caused an increase in cleavage of caspase 3 and poly-ADP ribose polymerase (PARP), indicating that more cells underwent apoptosis when pterostilbene was combined with megestrol acetate. Consistent with this result, an enhanced reduction of BCL-2 and BCL-xl were also found in cells treated with both pterostilbene and megestrol acetate (Fig. 3b).

In addition, we examined the effect of pterostilbene and megestrol acetate on cell cycle regulators, such as cyclin D1, cyclin B1 and CDK4. While pterostilbene alone inhibited the expression of these proteins in a dose dependent manner, megestrol acetate had little impact on these proteins (Fig. 3a). The combination treatment led to an increased inhibition of cyclin D1, cyclin B1 and CDK4 (Fig. 3b). Our results demonstrated that addition of pterostilbene to megestrol acetate led to an enhanced inhibition of cell survival and cell cycle progression in endometrial cancer.

### Combination treatment of pterostilbene and megestrol acetate suppresses ERK and STAT3 signaling pathways and estrogen receptor expression in endometrial cancer cells

**Figure 4:**
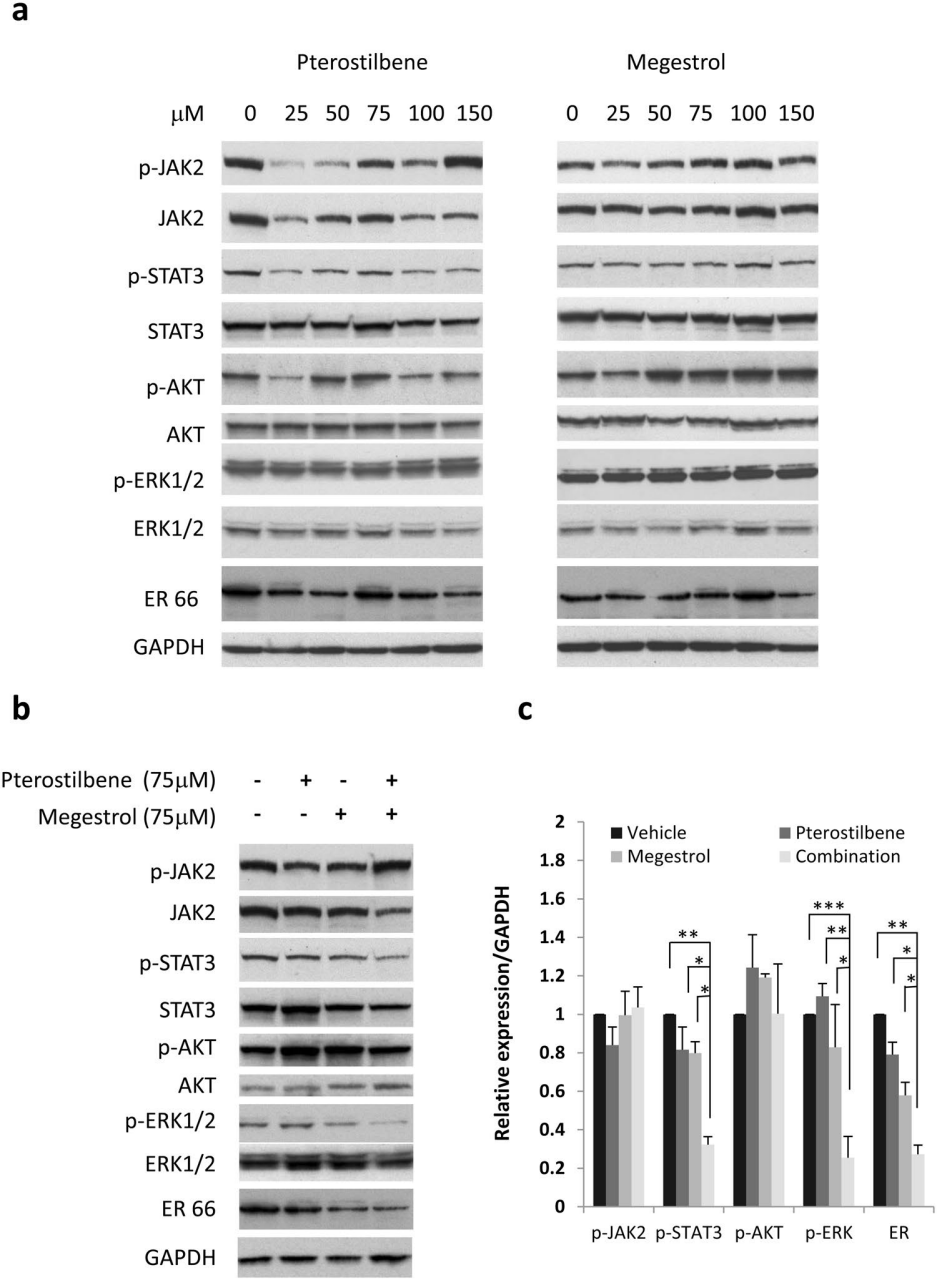
Effect of pterostilbene (PTE) and megestrol acetate (megestrol) on the expression of cell signaling molecules. (**a**) HEC-1A cells were treated with pterostilbene or megestrol acetate at various concentrations for 24 h. Whole cells were collected and determined for the change of STAT3, AKT and ERK pathways and ER expression by Western blot. (**b**) HEC-1A cells were treated with pterostilbene (75 μM), megestrol acetate (75 μM) or the combination for 24 h. Whole cell lysates were collected and measured for the change of cell signaling pathways by Western blot. Results are representative of 3 or more preparations. (**c**) Relative levels of p-JAK2, p-STAT3, p-AKT, p-ERK and ER were determined by measuring the density of each band and normalized to that of GAPDH. Densitometry data were relative changes in protein expression and were mean ± SD of more than 3 preparations. *P < 0.05; **P < 0.005, ***P < 0.005.

To understand the molecular mechanism underlying this synergistic effect, we investigated the molecular changes in the HEC-1A endometrial cancer cells in response to combination treatment of pterostilbene and megestrol acetate. A number of signaling pathways, including MAPK/ERK, PI3K/AKT and JAK/STAT3 pathways, are constitutively activated and play important roles in the growth and progression of endometrial cancer. To study the effect of pterostilbene and megestrol acetate on these signaling pathways, HEC-1A cells were treated with pterostilbene and megestrol acetate either alone or in combination for 24 hours, and tested for the expression of p-STAT3, p-AKT, p-ERK and ER-α by Western blot. As shown in Fig. 4a, treatment with pterostilbene or megestrol acetate alone had no effective inhibition on these pathways. However, the combined treatment with both pterostilbene and megestrol acetate led to the inhibition of p-STAT3, p-ERK and expression of ER-α (Fig. 4b,c). The inhibition of these signaling molecules by the combined treatment were considerably greater compared to any single treatment (Fig. 4b,c). Our results, therefore, demonstrated that dual treatment with pterostilbene and megestrol acetate can effectively inhibit multiple signaling pathways and led to an enhanced inhibition of cancer cell growth.

### Pterostilbene in combination with megestrol acetate reduces tumor growth in an endometrial cancer xenograft mouse model

**Figure 5:**
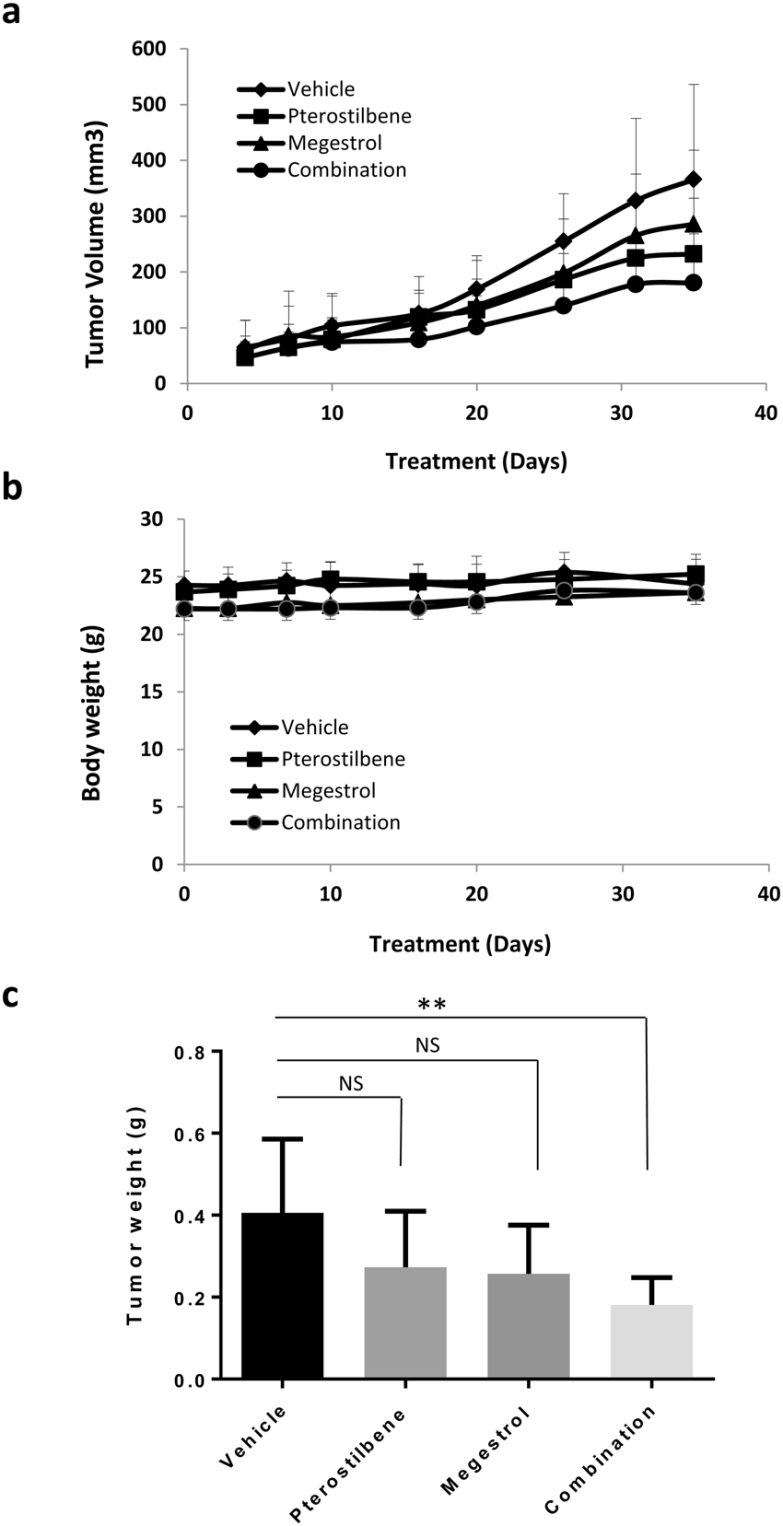
Anti-tumor activity of pterotilbene (PTE) plus megestrol acetate (megestrol) in HEC-1A xenograft model. HEC-1A cells were implanted subcutaneously into the right flank of nude mice. Tumors were treated daily by oral gavage with vehicle, Pterostilbene (30 mg/kg), megestrol acetate (10 mg/kg) or combination of both. (**a**) Tumor volume and (**b**) body weight were measured once or twice a week. (**c**) Tumor weight was measured at end of the treatment. Data represent means ± SD (n = 8–10). **P < 0.005, combination versus vehicle.

Anti-tumor activity of pterostilbene and/or megestrol acetate was evaluated in a HEC-1A xenograft mouse model (Fig. 5). HEC-1A cells were implanted subcutaneously in the right flank of nude mice. When the tumors are palpable, mice were randomized into four groups and treated with vehicle control, pterostilbene, megestrol acetate and pterostilbene plus megestrol acetate via oral gavage. Tumor volume (Fig. 5a) and body weight (Fig. 5b) were monitored twice weekly, and tumor weight measured at the end of the treatment (5 weeks) (Fig. 5c). The combination of pterostilbene and megestrol acetate showed significant tumor growth reduction (both tumor volume and tumor weight), while the tumor growth reduction for pterostilbene or megestrol acetate alone were non-significant (Fig. 5a,c).

## Discussion

Few treatment options are available for patients with advanced stage and recurrent endometrial carcinoma. Novel therapies are difficult to establish in this patient population which tends to be older and plagued by comorbidities such as diabetes mellitus, morbid obesity, and hypertension. Therefore, novel, non-toxic therapies are urgently needed. In the current investigation, the therapeutic effect of the addition of the orally available, natural antioxidant, pterostilbene, to a well established endometrial cancer therapy, megestrol acetate, was tested in endometrial cancer cells and a mouse model. Our results demonstrate for the first time that dual treatment with pterostilbene and megestrol acetate results in a synergistic anti-proliferative effect in endometrial cancer cells, and significantly reduces tumor growth in a xenograft endometrial cancer mouse model, as demonstrated by reduction in tumor weight and volume. Investigation into molecular mechanisms leading to this synergy reveals that the combination more effectively suppresses activation of STAT3 and ERK1/2 pathway, as well as ER expression, but did not impact AKT activation.

The role of pterostilbene in induction of apoptosis and cell cycle arrest has been demonstrated in other cancers, including bladder, lung and gastric cancer^32–34^. These pro-apoptotic effects have been observed in numerous *in vitro* tumor cell lines, and include up-regulation of proapoptotic mitochondrial derived proteins (BAX, BAK etc), while down-regulating anti-apoptotic proteins BCL-2 and BCL-xl, and inducing the expression of caspase 3^40^. For example, in breast cancer, pterostilbene induces apoptosis and anti-proliferation in ER-α rich breast cancer cells, with additive effect by administration of tamoxifen^41,42^. In endometrial cancer cell lines, pterostilbene was recently demonstrated to induce cytotoxicity via caspase-dependent apoptosis, via down-regulation of miR-663b, and up-regulation of BCL-G^43^. Our investigation demonstrated that pterostilbene as a single treatment led to an increased cleavage of an apoptotic marker, caspase 3, and a decreased expression of cell survival proteins, BCL-2 and BCL-xl, in endometrial cells, similar to the pro-apoptotic effects by pterostilbene reported for other cancer cell lines. In addition, pterostilbene alone inhibited the expression of the cell cycle regulators, such as cyclin D1, cyclin B1 and CDK4. These activities were further enhanced when pterostilbene was combined with megestrol acetate, while megestrol acetate alone had little effect on apoptosis and cell cycle progression in endometrial cells.

The effect of pterostilbene on estrogen receptors (ER) in endometrial cancer has not yet been studied elsewhere. Its structural analogue, resveratrol, has been shown to bind to both estrogen receptor alpha and beta^44–46^. Recently, both resveratrol and pterostilbene have been shown to act as ER beta agonists in prostate cancer cells, through which they inhibit cell proliferation via induction of mitochondrial antioxidant enzymes^47^. Combination of resveratrol and pterostilbene was also shown to restore ERalpha expression in triple negative breast cancer^48^. In colon cancer, pterostilbene suppressed AKT and ERK phosphorylation more effectively in ER-β rich colon cancer cells, as opposed to ER-β poor cells^35^. While the majority of ERs are located in the nucleus and act as transcription factors, estrogen binding to membranous ERs leads to non-genomic processes which activate signal transduction pathways such as PI3K/AKT and MAPK/ERK pathways^49^. This non-genomic effect has previously been described in the endometrial cancer cell line, HEC-1A, where estradiol binding to ER induces ERK1/2 activation, but not AKT activation^38^. Similarly, our results show that in HEC-1A cells, the combination of pterostilbene and megestrol acetate suppresses ERK1/2 phosphorylation, but not AKT phosphorylation. Whether binding of pterostilbene to membrane-bound ERs reduces estrogen binding and therefore results in attenuation of the MAPK/ERK pathway is unknown, but may be hypothesized from the above studies. Our study revealed a mild reduction of ER expression by pterostilbene or megestrol acetate alone, however the combination of megestrol acetate and pterostilbene significantly reduced ER expression in endometrial cancer cells. Conventionally speaking, progestins are not anti-estrogens, and only indirectly reduce ER expression via negative feedback. Despite decades of use, the exact mechanism of progestin therapy has not been elucidated in endometrial cancer, and has been attributed largely to the atrophy-inducing effect on the endometrium^16,50–52^. Nonetheless, lung metastases from endometrial cancer can occasionally be treated effectively with progestins. In our study, neither pterostilbene nor megestrol acetate alone substantially attenuated ERK signaling, though their combined effect on the attenuation of the ERK pathway points to an additive effect in enhanced antineoplastic activity. One could hypothesize that the combination of megestrol acetate and pterostilbene is synergistic in inhibiting important signaling pathways such as MAPK/ERK, by way of reduced ER expression via megestrol acetate, and ER binding by pterostilbene, but further studies are needed to confirm this.

Our *in vivo* study demonstrates that dual treatment with pterostilbene and megestrol acetate results in significant tumor growth inhibition. The pterostilbene dose used for oral gavage of mice was 30 mg/kg, which is reported to be the equivalent of 5 times the mean human intake of pterostilbene (25 mg/day), or 125 mg/day for humans^53^. A recent clinical trial investigating the safety of pterostilbene concluded that pterostilbene is generally safe for use in humans up to 250 mg/day^54^. Similarly, the megestrol acetate dose used in our animal study (10 mg/kg), would favorably compare to human doses of up to 800 mg/day used for endometrial cancer patients^13^. Both pterostilbene and megestrol acetate are well tolerated in comparison to most cytotoxic treatments for endometrial cancer, and its synergistic *in vivo* activity to inhibit tumor growth is thus promising for endometrial cancer patients who frequently have a poor performance status.

Our study shows that the dual treatment with pterostilbene and megestrol acetate can inhibit multiple cell growth and survival pathways and results in an enhanced inhibition of cancer cell growth. The above results show promising anti-tumor activity of the combination of pterostilbene and megestrol acetate in endometrial cancer, and deserve further study.

## Materials and Methods

### Reagents

Pterostilbene was kindly provided by Chromadex, Inc, Irvine, CA. Megestrol acetate was from Selleck Chemicals (Houston, TX), megestrol acetate suspension was obtained from Morton Grove Pharmaceuticals (Morton Grove, IL). Antibodies against p-ERK (T202/Y204), ERK, p-STAT3, STAT3, p-JAK2, JAK2, p-AKT (S473), BCL-2, BCL-XL, caspase 3, PARP, ER-66 and GAPDH were obtained from Cell Signaling Technology (Danvers, MA). The antibody against cyclin B1 was from BD Bioscience (San Jose, CA). The antibody against cyclin D1 and CDK4 were from Thermo Scientific (Carlsbad, CA). The antibody against AKT was from Santa Cruz Biotechnology (Dallas, TX).

### Cell Culture

Human endometrial cancer cell lines HEC-1A and ECC-1 cells were from American Type Culture Collection (Rockville, MD). Both cell lines were cultured in RPMI-1640 medium, containing 10% FBS and 1% penicillin/streptomycin (P/S). All cells were grown in 5% (v/v) CO_2_ at 37 °C.

### Cell viability assays

Cells (4000 per well) were plated in 96-well plate format in 100 μl growth medium. Cells were treated with DMSO or drugs the next day at the indicated concentrations and incubated for an additional 2–3 days. Viable cells were determined either by the MTS assay (Promega, Madison, WI, USA) or the acid phosphatase assay^55,56^. For the MTS assay, 25 μl MTS solution was added directly into each well according to the manufacturer’s instructions. For the acid phophatase assay, all the media was removed and p-nitrophenyl phosphate substrate (10 mM 100μl) was added into each well and incubated at 37 °C for 45 mins. NaOH was added to stop the reaction and the absorbance was read at 415 nM. The IC_50_ was determined using the Calcusyn software (Biosoft, Ferguson, MO).

### Determination of combination index (CI)

The combination index (CI) was determined using the Chou-Talalay method^39^ using the Calcusyn software (Biosoft, MO).

### Western blot analysis

Western blots were performed as described previously^57,58^. Cells were grown in complete medium overnight and treated with DMSO or drugs at various concentrations for 24 hrs. Cells were washed in cold PBS and lysed in RIPA lysis buffer (Thermo Scientific) containing Halt protease and phosphatase inhibitors (Thermo Scientific). Proteins were quantified using BCA protein assay reagent (Thermo Scientific). Equal amounts of protein were separated by SDS-polyacrylamide gel electrophoresis, transferred to polyvinylidene fluoride membranes and incubated with total and phosphorylated protein-specific antibodies. Binding of the primary antibody was detected using a horseradish peroxidase (HRP)-conjugated secondary antibody and chemiluminescent substrates (Thermo Scientific).

### Animal models

All animal studies were carried out under protocols approved by the Institutional Animal Care and Use Committee (IACUC) at City of Hope in accordance with all applicable federal, state, and local requirements and institutional guidelines. HEC-1A cells (2 × 10^6^ in 100 μl) were inoculated subcutaneously into the right flank of 6- to 8- week-old female nude mice. Once the tumors were palpable, animals were randomized into groups of 10 to achieve an equal distribution of tumor sizes in all treatment groups. Mice were then treated by oral gavage daily with vehicle, pterostilbene (30 mg/kg), megestrol acetate (10 mg/kg), or a combination of both agents. The doses for these two drugs were chosen based on previously published studies. Tumor volumes were assessed using calipers twice a week. Tumor volumes were determined using the formula (Width)^2^ × Length × 0.52. Body weight was monitored weekly as an indicator of drug-induced toxicity and overall health of the mice.

### Statistical analysis

Data are presented as mean ± S.D. Student’s t-test was used to compare the means of two groups. All the experiments were carried out in triplicate or more. P < 0.05 was considered statistically significant.

## References

[1] Siegel RL, Miller KD, Jemal A (2016). Cancer statistics, 2016. CA Cancer J Clin.

[2] Morice P, Leary A, Creutzberg C, Abu-Rustum N, Darai E (2016). Endometrial cancer. Lancet.

[3] Dizon DS, Birrer MJ (2014). Advances in the diagnosis and treatment of uterine sarcomas. Discovery medicine.

[4] Oza AM (2011). Phase II study of temsirolimus in women with recurrent or metastatic endometrial cancer: a trial of the NCIC Clinical Trials Group. J Clin Oncol.

[5] Aghajanian C (2011). Phase II trial of bevacizumab in recurrent or persistent endometrial cancer: a Gynecologic Oncology Group study. J Clin Oncol.

[6] Burke WM (2014). Endometrial cancer: A review and current management strategies: Part II. Gynecologic Oncology.

[7] Burke WM (2014). Endometrial cancer: A review and current management strategies: Part I. Gynecologic Oncology.

[8] Bradford LS, Rauh-Hain JA, Schorge J, Birrer MJ, Dizon DS (2015). Advances in the management of recurrent endometrial cancer. American journal of clinical oncology.

[9] Lheureux, S. & Oza, A. M. Endometrial cancer—targeted therapies myth or reality? Review of current targeted treatments. *European Journal of Cancer* **59**, 99–108, 10.1016/j.ejca.2016.02.016 (2016). 10.1016/j.ejca.2016.02.016 27017291

[10] Myers AP (2016). Tumor mutational analysis of GOG248, a phase II study of temsirolimus or temsirolimus and alternating megestrol acetate and tamoxifen for advanced endometrial cancer (EC): An NRG Oncology/Gynecologic Oncology Group study. Gynecologic Oncology.

[11] DeLeon MC, Ammakkanavar NR, Matei D (2014). Adjuvant therapy for endometrial cancer. Journal of Gynecologic Oncology.

[12] Hansen J (2015). The effect of weight-based chemotherapy dosing in a cohort of gynecologic oncology patients. Gynecologic Oncology.

[13] Lentz SS, Brady MF, Major FJ, Reid GC, Soper JT (1996). High-dose megestrol acetate in advanced or recurrent endometrial carcinoma: a Gynecologic Oncology Group Study. J Clin Oncol.

[14] Rauh-Hain JA, del Carmen MG (2010). Treatment for Advanced and Recurrent Endometrial Carcinoma: Combined Modalities. The Oncologist.

[15] Lee WL (2014). Hormone therapy for patients with advanced or recurrent endometrial cancer. Journal of the Chinese Medical Association: JCMA.

[16] Yang S, Thiel KW, De Geest K, Leslie KK (2011). Endometrial cancer: reviving progesterone therapy in the molecular age. Discovery medicine.

[17] Kong Y (2016). Pterostilbene induces apoptosis and cell cycle arrest in diffuse large B-cell lymphoma cells. Scientific reports.

[18] Lee H, Kim Y, Jeong JH, Ryu J-H, Kim W-Y (2016). ATM/CHK/p53 Pathway Dependent Chemopreventive and Therapeutic Activity on Lung Cancer by Pterostilbene. PLoS ONE.

[19] Dhar S (2016). Dietary pterostilbene is a novel MTA1-targeted chemopreventive and therapeutic agent in prostate cancer. Oncotarget.

[20] Nikhil K, Sharan S, Singh AK, Chakraborty A, Roy P (2014). Anticancer Activities of Pterostilbene-Isothiocyanate Conjugate in Breast Cancer Cells: Involvement of PPARγ. PLoS ONE.

[21] Li K (2013). Pterostilbene Acts through Metastasis-Associated Protein 1 to Inhibit Tumor Growth, Progression and Metastasis in Prostate Cancer. PLoS ONE.

[22] Paul S (2009). Anti-inflammatory action of pterostilbene is mediated through the p38 MAPK pathway in colon cancer cells. Cancer prevention research (Philadelphia, Pa.).

[23] Pan M-H (2009). Pterostilbene inhibited tumor invasion via suppressing multiple signal transduction pathways in human hepatocellular carcinoma cells. Carcinogenesis.

[24] Suh N (2007). Pterostilbene, an Active Constituent of Blueberries, Suppresses Aberrant Crypt Foci Formation in the Azoxymethane-Induced Colon Carcinogenesis Model in Rats. Clinical Cancer Research.

[25] Hsiao PC (2014). Pterostilbene simultaneously induced G0/G1-phase arrest and MAPK-mediated mitochondrial-derived apoptosis in human acute myeloid leukemia cell lines. PLoS ONE.

[26] Priego S (2008). Natural polyphenols facilitate elimination of HT-29 colorectal cancer xenografts by chemoradiotherapy: a Bcl-2- and superoxide dismutase 2-dependent mechanism. Molecular Cancer Therapeutics.

[27] Xie B (2016). Pterostilbene Inhibits Human Multiple Myeloma Cells via ERK1/2 and JNK Pathway *In Vitro* and *In Vivo*. International Journal of Molecular Sciences.

[28] Schmidt L (2016). Case-specific potentiation of glioblastoma drugs by pterostilbene. Oncotarget.

[29] Rimando AM (2002). Cancer Chemopreventive and Antioxidant Activities of Pterostilbene, a Naturally Occurring Analogue of Resveratrol. Journal of Agricultural and Food Chemistry.

[30] Estrela, J. M. *et al*. Polyphenolic Phytochemicals in Cancer Prevention and Therapy: Bioavailability versus Bioefficacy. *J Med Chem*, 10.1021/acs.jmedchem.6b01026 (2017). 10.1021/acs.jmedchem.6b01026 28654265

[31] Estrela JM, Ortega A, Mena S, Rodriguez ML, Asensi M (2013). Pterostilbene: Biomedical applications. Critical reviews in clinical laboratory sciences.

[32] Pan C (2014). Estrogen receptor-alpha36 is involved in pterostilbene-induced apoptosis and anti-proliferation in *in vitro* and *in vivo* breast cancer. PLoS ONE.

[33] Chen RJ, Ho CT, Wang YJ (2010). Pterostilbene induces autophagy and apoptosis in sensitive and chemoresistant human bladder cancer cells. Mol Nutr Food Res.

[34] Schneider JG, Alosi JA, McDonald DE, McFadden DW (2010). Pterostilbene inhibits lung cancer through induction of apoptosis. The Journal of surgical research.

[35] Tolba MF, Abdel-Rahman SZ (2015). Pterostilbine, an active component of blueberries, sensitizes colon cancer cells to 5-fluorouracil cytotoxicity. Scientific reports.

[36] Kuramoto H (1972). Studies of the growth and cytogenetic properties of human endometrial adenocarcinoma in culture and its development into an established line. Acta obstetrica et gynaecologica Japonica.

[37] Mo B (2006). ECC-1 cells: a well-differentiated steroid-responsive endometrial cell line with characteristics of luminal epithelium. Biology of reproduction.

[38] Zhang L (2009). Nongenomic effect of estrogen on the MAPK signaling pathway and calcium influx in endometrial carcinoma cells. J Cell Biochem.

[39] Chou, T.-C. Drug Combination Studies and Their Synergy Quantification Using the Chou-Talalay Method. *Cancer Res* **70**, 440-446, 10.1158/0008-5472.can-09-1947. 10.1158/0008-5472.CAN-09-1947 20068163

[40] Kosuru R, Rai U, Prakash S, Singh A, Singh S (2016). Promising therapeutic potential of pterostilbene and its mechanistic insight based on preclinical evidence. European journal of pharmacology.

[41] Mannal P, McDonald D, McFadden D (2010). Pterostilbene and tamoxifen show an additive effect against breast cancer *in vitro*. American journal of surgery.

[42] Mannal PW, Alosi JA, Schneider JG, McDonald DE, McFadden DW (2010). Pterostilbene inhibits pancreatic cancer *in vitro*. Journal of gastrointestinal surgery: official journal of the Society for Surgery of the Alimentary Tract.

[43] Wang, Y. L. *et al*. Pterostilbene suppresses human endometrial cancer cells *in vitro* by down-regulating miR-663b. *Acta pharmacologica Sinica*, 10.1038/aps.2017.60 (2017). 10.1038/aps.2017.60 PMC5630673 28552912

[44] Gehm BD, McAndrews JM, Chien PY, Jameson JL (1997). Resveratrol, a polyphenolic compound found in grapes and wine, is an agonist for the estrogen receptor. Proc Natl Acad Sci USA.

[45] Bowers JL, Tyulmenkov VV, Jernigan SC, Klinge CM (2000). Resveratrol acts as a mixed agonist/antagonist for estrogen receptors alpha and beta. Endocrinology.

[46] Bhat KP (2001). Estrogenic and antiestrogenic properties of resveratrol in mammary tumor models. Cancer Res.

[47] Robb EL, Stuart JA (2014). The stilbenes resveratrol, pterostilbene and piceid affect growth and stress resistance in mammalian cells via a mechanism requiring estrogen receptor beta and the induction of Mn-superoxide dismutase. Phytochemistry.

[48] Kala R, Tollefsbol TO (2016). A Novel Combinatorial Epigenetic Therapy Using Resveratrol and Pterostilbene for Restoring Estrogen Receptor-α (ERα) Expression in ERα-Negative Breast Cancer Cells. PLoS ONE.

[49] Bjornstrom L, Sjoberg M (2004). Estrogen receptor-dependent activation of AP-1 via non-genomic signalling. Nuclear receptor.

[50] Kim JJ, Kurita T, Bulun SE (2013). Progesterone Action in Endometrial Cancer, Endometriosis, Uterine Fibroids, and Breast Cancer. Endocrine Reviews.

[51] Dai D, Wolf DM, Litman ES, White MJ, Leslie KK (2002). Progesterone Inhibits Human Endometrial Cancer Cell Growth and Invasiveness. Cancer Research.

[52] Zhang K, Chow PK (2004). The effect of megestrol acetate on growth of HepG2 cells *in vitro* and *in vivo*. Clin Cancer Res.

[53] Ruiz MJ (2009). Dietary administration of high doses of pterostilbene and quercetin to mice is not toxic. Journal of agricultural and food chemistry.

[54] Riche DM (2013). Analysis of safety from a human clinical trial with pterostilbene. Journal of toxicology.

[55] Yang TT, Sinai P, Kain SR (1996). An acid phosphatase assay for quantifying the growth of adherent and nonadherent cells. Analytical biochemistry.

[56] Wen W (2015). Synergistic anti-tumor effect of combined inhibition of EGFR and JAK/STAT3 pathways in human ovarian cancer. Mol Cancer.

[57] Wen W (2014). Targeting JAK1/STAT3 signaling suppresses tumor progression and metastasis in a peritoneal model of human ovarian cancer. Mol Cancer Ther.

[58] Lu J (2010). Novel angiogenesis inhibitory activity in cinnamon extract blocks VEGFR2 kinase and downstream signaling. Carcinogenesis.

